# Bis­(3,5-diamino-4*H*-1,2,4-triazol-1-ium) 3,4-dioxocyclo­butane-1,2-diolate

**DOI:** 10.1107/S160053681300322X

**Published:** 2013-02-06

**Authors:** Hoong-Kun Fun, Wan-Sin Loh, Atim Johnson, Sammer Yousuf, Ededet Eno

**Affiliations:** a X-ray Crystallography Unit, School of Physics, Universiti Sains Malaysia, 11800 USM, Penang, Malaysia; b Department of Pharmaceutical Chemistry, College of Pharmacy, King Saud University, PO Box 2457, Riyadh 11451, Saudi Arabia; c H. E. J Research Institute of Chemistry, International Center for Chemical and Biological Sciences, University of Karachi, Karachi, 75720, Pakistan; d Department of Pure and Applied Chemistry, University of Calabar, P. M. B. 1115, Calabar, Nigeria

## Abstract

The asymmetric unit of the title compound, 2C_2_H_6_N_5_^+^·C_4_O_4_^2−^, contains two 3,5-diamino-4*H*-1,2,4-triazolium cations and one squarate dianion. The squaric acid mol­ecule donated one H atom to each of the two 3,5-diamino-1,2,4-triazole mol­ecules at their N atoms. The squaric acid dianion has four C—O bonds which are shorter than a normal single C—O bond (1.426 Å) and are slightly longer than a normal C=O bond (1.23 Å), which indicates the degree of electron delocalization in the dianion. In the crystal, the cations and dianions are linked by N—H⋯N and N—H⋯O hydrogen bonds into a three-dimensional network.

## Related literature

For background to the acid–base chemistry of squarate acid, see: Mathew *et al.* (2002 ▶); Frankenbach *et al.* (1992 ▶); Yeşilel *et al.* (2008 ▶); Bertolasi *et al.* (2001 ▶); Correa *et al.* (2007 ▶). For a related structure, see: Uçar *et al.* (2004 ▶). For the stability of the temperature controller used for the data collection, see: Cosier & Glazer (1986 ▶).
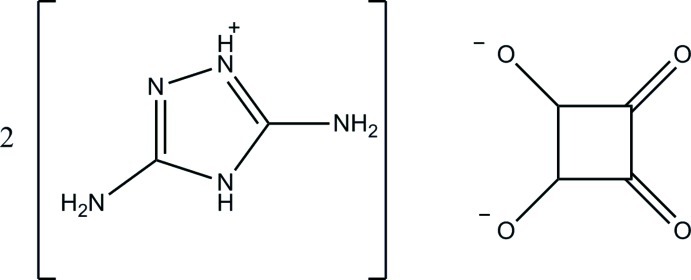


## Experimental

### 

#### Crystal data

2C_2_H_6_N_5_^+^·C_4_O_4_^2−^
*M* *_r_* = 312.28
Monoclinic, 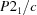

*a* = 15.7186 (2) Å
*b* = 11.6533 (2) Å
*c* = 6.8618 (1) Å
β = 91.734 (1)°
*V* = 1256.32 (3) Å^3^
*Z* = 4
Mo *K*α radiation
μ = 0.14 mm^−1^
*T* = 100 K
0.44 × 0.20 × 0.14 mm

#### Data collection

Bruker SMART APEXII CCD area-detector diffractometer
Absorption correction: multi-scan (*SADABS*; Bruker, 2009 ▶) *T*_min_ = 0.943, *T*_max_ = 0.982
19113 measured reflections
4965 independent reflections
3911 reflections with *I* > 2σ(*I*)
*R*_int_ = 0.028

#### Refinement

*R*[*F* ^2^ > 2σ(*F* ^2^)] = 0.040
*wR*(*F* ^2^) = 0.108
*S* = 1.03
4965 reflections
247 parameters
All H-atom parameters refined
Δρ_max_ = 0.43 e Å^−3^
Δρ_min_ = −0.32 e Å^−3^

### 

Data collection: *APEX2* (Bruker, 2009 ▶); cell refinement: *SAINT* (Bruker, 2009 ▶); data reduction: *SAINT*; program(s) used to solve structure: *SHELXTL* (Sheldrick, 2008 ▶); program(s) used to refine structure: *SHELXTL*; molecular graphics: *SHELXTL*; software used to prepare material for publication: *SHELXTL* and *PLATON* (Spek, 2009 ▶).

## Supplementary Material

Crystal structure: contains datablock(s) global, I. DOI: 10.1107/S160053681300322X/rz5040sup1.cif

Structure factors: contains datablock(s) I. DOI: 10.1107/S160053681300322X/rz5040Isup2.hkl

Supplementary material file. DOI: 10.1107/S160053681300322X/rz5040Isup3.cml

Additional supplementary materials:  crystallographic information; 3D view; checkCIF report

## Figures and Tables

**Table 1:** Hydrogen-bond geometry (Å, °)

*D*—H⋯*A*	*D*—H	H⋯*A*	*D*⋯*A*	*D*—H⋯*A*
N1*A*—H1*N*1⋯O4^i^	0.913 (16)	1.761 (16)	2.6677 (9)	171.3 (15)
N3*A*—H1*N*3⋯O4^ii^	0.937 (15)	1.746 (15)	2.6734 (10)	170.2 (14)
N4*A*—H1*N*4⋯O3^i^	0.887 (15)	2.003 (15)	2.8877 (10)	175.2 (13)
N4*A*—H2*N*4⋯N4*B* ^iii^	0.904 (15)	2.565 (15)	3.3917 (12)	152.3 (12)
N5*A*—H1*N*5⋯N2*A* ^iv^	0.933 (15)	2.105 (15)	3.0167 (11)	165.3 (13)
N5*A*—H2*N*5⋯O1^ii^	0.922 (15)	1.940 (15)	2.8621 (11)	177.7 (14)
N1*B*—H2*N*1⋯O2^v^	0.874 (15)	1.781 (15)	2.6485 (9)	171.8 (15)
N3*B*—H2*N*3⋯O2	0.965 (15)	1.706 (15)	2.6637 (10)	171.2 (14)
N4*B*—H3*N*4⋯O1^v^	0.920 (14)	2.065 (14)	2.9564 (10)	162.8 (12)
N4*B*—H4*N*4⋯O1^vi^	0.867 (13)	2.150 (13)	2.9954 (10)	164.9 (12)
N5*B*—H3*N*5⋯N2*B* ^v^	0.923 (15)	2.159 (15)	3.0579 (11)	164.3 (12)
N5*B*—H4*N*5⋯O3	1.001 (16)	1.832 (15)	2.8293 (10)	174.2 (12)

Symmetry codes: (i) 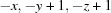; (ii) 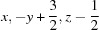; (iii) 
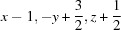
; (iv) 
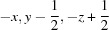
; (v) 
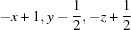
; (vi) 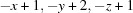.

**Fig. 1.**
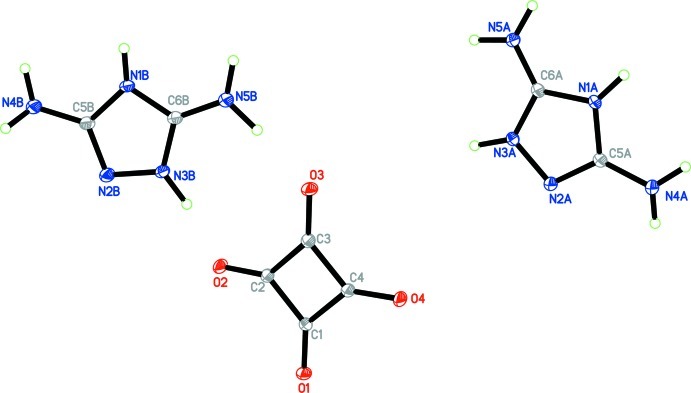
The molecular structure of the title compound, showing 50% probability displacement ellipsoids.

**Fig. 2.**
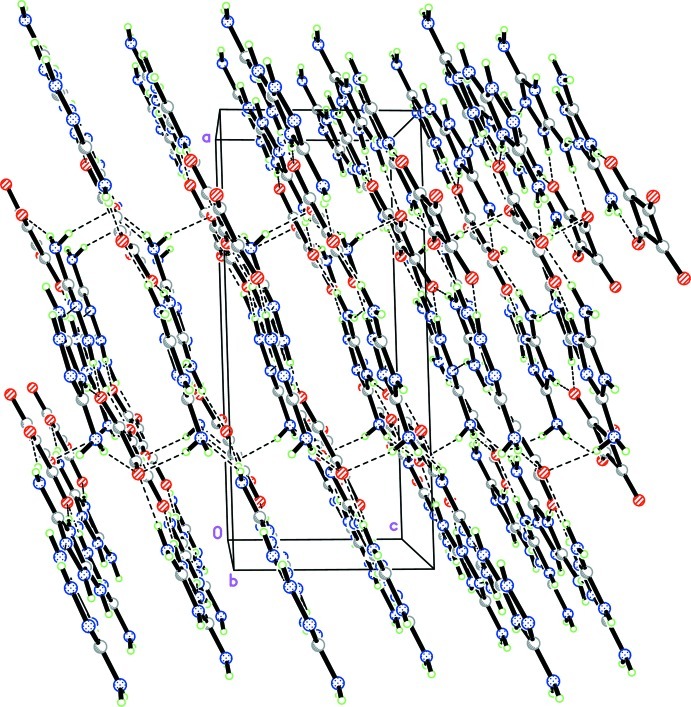
The crystal packing of the title compound, approximately viewed along the b axis, showing the three dimensional network.

## supplementary crystallographic information

### Comment

Supramolecularly organized systems with a variety of novel features are widely generated through hydrogen-bonding. Hydrogen-bonded systems generated from organic cations and anions are of special interest since they are likely to show stronger hydrogen bonds than neutral molecules thus enabling the simple acid-base chemistry to tune the donor and acceptor properties of the counter ions (Mathew *et al.*, 2002). Squaric acid (H_2_C_4_O_4_, 3,4-dihydroxy-3-cyclobutene-1,2-dione) has been of much interests because of its cyclic structure and possible aromaticity (Frankenbach *et al.*, 1992; Yeşilel *et al.*, 2008). The molecule possesses a certain degree of electron delocalization, but it is most pronounced in the dianion (Mathew *et al.* 2002). This property is important in crystal packing (Bertolasi *et al.*, 2001). The squarate dianion does not act like a chelating ligand but rather like a bridge between two or more metal atoms as a mono- or polydentate ligand. The 1,3-bis (monodentate) bridging coordination mode is very useful in generating one dimensional polymeric structures and the dimensionality can be expanded to two-dimensional or three-dimensional arrays using multidentate spacer ligands (Correa *et al.*, 2007). We have been interested in the preparation of metal complexes by organic amines and carboxylic acids. In line with our interests, it was our design to synthesize a squarato-bridged zinc(II) complex. However, our proposed structure was not obtained; instead a new polymeric supramolecular triazolium squarate structure was formed. Herein we present the crystal structure of the new compound.

The asymmetric unit of the title compound, Fig. 1, contains two 3,5-diamino-4*H*-1,2,4-triazolium cations (C5/C6/N1–N5) and one squarate dianion (C1–C4/O1–O4). The squaric acid molecule donates one proton to each of the 3,5-diamino-1,2,4-triazole at N3A and N3B atoms which result in the formation of the 3,5-diamino-4*H*-1,2,4-triazolium squarate salt. The squaric acid dianion has four C–O bonds [C1—O1 = 1.2599 (10) Å, C2—O2 = 1.2608 (10) Å, C3—O3 = 1.2490 (10) Å, C4—O4 = 1.2622 (10) Å] which are shorter than normal single C—O bond (1.426 Å). These bonds, however, are slightly longer than normal C═O bond (1.23 Å). These bond lengths are indicative of the degree of electron delocalization in the dianion (Mathew *et al.* 2002; Bertolasi *et al.*, 2001; Uçar *et al.*, 2004).

In the crystal packing (Fig. 2), the structure of the compound is stabilized by intermolecular N—H···N and N—H···O hydrogen bonds (Table 1) into a three dimensional network.

### Experimental

Zinc chloride (1 mmol, 0.136 g) and 3,5-diamino-1,2,4-triazole (1 mmol, 0.099 g) were dissolved in 10 ml of distilled water. The solution was heated gently for 5 minutes, followed by drop-wise addition of an aqueous solution of squaric acid (0.057 g, 0.5 mmol) dissolved in 5 ml of hot water. The mixture was heated on a steam bath for 15 minutes and filtered while hot. The filtrate was allowed to crystallize at ambient temperature. The compound crystallized out after two weeks. CHN-analysis: found, C, 30.79; H, 3.82; N, 44.89; calcd. for C_8_H_12_N_10_O_4_: C, 30.76; H, 3.85; N, 44.93.

### Refinement

All the H atoms were located in a difference Fourier map and were refined freely [N–H = 0.867 (13) to 1.001 (15) Å].

### Crystal data

**Table:**

2C_2_H_6_N_5_^+^·C_4_O_4_^2^^−^	*F*(000) = 648
*M**_r_* = 312.28	*D*_x_ = 1.651 Mg m^−^^3^
Monoclinic, *P*2_1_/*c*	Mo *K*α radiation, λ = 0.71073 Å
Hall symbol: -P 2ybc	Cell parameters from 7050 reflections
*a* = 15.7186 (2) Å	θ = 2.2–33.7°
*b* = 11.6533 (2) Å	µ = 0.14 mm^−^^1^
*c* = 6.8618 (1) Å	*T* = 100 K
β = 91.734 (1)°	Block, colourless
*V* = 1256.32 (3) Å^3^	0.44 × 0.20 × 0.14 mm
*Z* = 4	

### Data collection

**Table:**

Bruker SMART APEXII CCD area-detector diffractometer	4965 independent reflections
Radiation source: fine-focus sealed tube	3911 reflections with *I* > 2σ(*I*)
Graphite monochromator	*R*_int_ = 0.028
φ and ω scans	θ_max_ = 33.7°, θ_min_ = 1.3°
Absorption correction: multi-scan (*SADABS*; Bruker, 2009)	*h* = −24→23
*T*_min_ = 0.943, *T*_max_ = 0.982	*k* = −18→12
19113 measured reflections	*l* = −10→10

### Refinement

**Table:**

Refinement on *F*^2^	Primary atom site location: structure-invariant direct methods
Least-squares matrix: full	Secondary atom site location: difference Fourier map
*R*[*F*^2^ > 2σ(*F*^2^)] = 0.040	Hydrogen site location: inferred from neighbouring sites
*wR*(*F*^2^) = 0.108	All H-atom parameters refined
*S* = 1.03	*w* = 1/[σ^2^(*F*_o_^2^) + (0.0597*P*)^2^ + 0.1328*P*] where *P* = (*F*_o_^2^ + 2*F*_c_^2^)/3
4965 reflections	(Δ/σ)_max_ = 0.001
247 parameters	Δρ_max_ = 0.43 e Å^−^^3^
0 restraints	Δρ_min_ = −0.32 e Å^−^^3^

### Special details

**Table:**

Experimental. The crystal was placed in the cold stream of an Oxford Cryosystems Cobra open-flow nitrogen cryostat (Cosier & Glazer, 1986) operating at 100.0 (1) K.
Geometry. All e.s.d.'s (except the e.s.d. in the dihedral angle between two l.s. planes) are estimated using the full covariance matrix. The cell e.s.d.'s are taken into account individually in the estimation of e.s.d.'s in distances, angles and torsion angles; correlations between e.s.d.'s in cell parameters are only used when they are defined by crystal symmetry. An approximate (isotropic) treatment of cell e.s.d.'s is used for estimating e.s.d.'s involving l.s. planes.
Refinement. Refinement of *F*^2^ against ALL reflections. The weighted *R*-factor *wR* and goodness of fit *S* are based on *F*^2^, conventional *R*-factors *R* are based on *F*, with *F* set to zero for negative *F*^2^. The threshold expression of *F*^2^ > σ(*F*^2^) is used only for calculating *R*-factors(gt) *etc*. and is not relevant to the choice of reflections for refinement. *R*-factors based on *F*^2^ are statistically about twice as large as those based on *F*, and *R*- factors based on ALL data will be even larger.

### Fractional atomic coordinates and isotropic or equivalent isotropic displacement parameters (Å^2^)

**Table:**

	*x*	*y*	*z*	*U*_iso_*/*U*_eq_	
O1	0.21331 (4)	1.07699 (5)	0.54430 (10)	0.01452 (13)	
O2	0.37416 (4)	0.94620 (5)	0.36357 (10)	0.01592 (14)	
O3	0.29531 (4)	0.70043 (5)	0.48973 (11)	0.01665 (14)	
O4	0.13065 (4)	0.82957 (5)	0.65486 (10)	0.01628 (14)	
C1	0.23528 (5)	0.97349 (7)	0.52898 (13)	0.01176 (15)	
C2	0.30810 (5)	0.91452 (7)	0.44836 (13)	0.01202 (15)	
C3	0.27269 (5)	0.80280 (7)	0.50302 (13)	0.01267 (16)	
C4	0.19890 (5)	0.86217 (7)	0.57988 (13)	0.01233 (16)	
N1A	−0.07849 (5)	0.38750 (6)	0.31736 (11)	0.01324 (15)	
N2A	−0.06499 (5)	0.57809 (6)	0.31893 (11)	0.01364 (15)	
N3A	0.00592 (5)	0.52443 (6)	0.23974 (12)	0.01388 (15)	
N4A	−0.19073 (5)	0.49920 (7)	0.44806 (13)	0.01773 (17)	
N5A	0.05462 (5)	0.33657 (7)	0.17618 (13)	0.01821 (17)	
C5A	−0.11444 (5)	0.49176 (7)	0.36330 (13)	0.01248 (16)	
C6A	−0.00190 (5)	0.41097 (7)	0.24079 (13)	0.01293 (16)	
N1B	0.58254 (5)	0.65997 (6)	0.21916 (11)	0.01269 (14)	
N2B	0.57382 (5)	0.84985 (6)	0.24748 (12)	0.01463 (15)	
N3B	0.49823 (5)	0.79658 (6)	0.29850 (12)	0.01433 (15)	
N4B	0.70576 (5)	0.77532 (7)	0.14462 (12)	0.01499 (15)	
N5B	0.44176 (5)	0.60870 (7)	0.31739 (13)	0.01730 (16)	
C5B	0.62343 (5)	0.76414 (7)	0.20276 (13)	0.01231 (16)	
C6B	0.50327 (5)	0.68311 (7)	0.27941 (13)	0.01274 (16)	
H1N1	−0.1018 (10)	0.3160 (14)	0.327 (2)	0.040 (4)*	
H1N3	0.0528 (9)	0.5685 (13)	0.204 (2)	0.032 (4)*	
H1N4	−0.2223 (9)	0.4367 (13)	0.460 (2)	0.037 (4)*	
H2N4	−0.2172 (9)	0.5679 (13)	0.456 (2)	0.033 (4)*	
H1N5	0.0480 (9)	0.2572 (13)	0.186 (2)	0.037 (4)*	
H2N5	0.1053 (10)	0.3664 (13)	0.134 (2)	0.039 (4)*	
H2N1	0.6019 (10)	0.5916 (13)	0.193 (2)	0.037 (4)*	
H2N3	0.4496 (9)	0.8444 (13)	0.323 (2)	0.037 (4)*	
H3N4	0.7334 (8)	0.7088 (12)	0.112 (2)	0.028 (3)*	
H4N4	0.7362 (8)	0.8208 (11)	0.2184 (19)	0.028 (3)*	
H3N5	0.4482 (9)	0.5311 (13)	0.295 (2)	0.039 (4)*	
H4N5	0.3877 (10)	0.6390 (14)	0.371 (2)	0.044 (4)*	

### Atomic displacement parameters (Å^2^)

**Table:**

	*U*^11^	*U*^22^	*U*^33^	*U*^12^	*U*^13^	*U*^23^
O1	0.0142 (3)	0.0081 (2)	0.0215 (3)	0.0007 (2)	0.0043 (2)	−0.0010 (2)
O2	0.0124 (3)	0.0102 (3)	0.0256 (4)	−0.0002 (2)	0.0086 (2)	0.0010 (2)
O3	0.0149 (3)	0.0082 (3)	0.0272 (4)	0.0016 (2)	0.0067 (2)	0.0004 (2)
O4	0.0124 (3)	0.0103 (3)	0.0267 (4)	−0.0010 (2)	0.0088 (2)	−0.0001 (2)
C1	0.0109 (3)	0.0092 (3)	0.0154 (4)	−0.0008 (3)	0.0023 (3)	−0.0004 (3)
C2	0.0105 (3)	0.0092 (3)	0.0165 (4)	−0.0001 (3)	0.0030 (3)	0.0002 (3)
C3	0.0115 (3)	0.0098 (3)	0.0168 (4)	−0.0001 (3)	0.0032 (3)	0.0005 (3)
C4	0.0115 (3)	0.0091 (3)	0.0166 (4)	−0.0002 (3)	0.0035 (3)	−0.0002 (3)
N1A	0.0128 (3)	0.0090 (3)	0.0182 (4)	−0.0020 (2)	0.0044 (3)	0.0001 (3)
N2A	0.0124 (3)	0.0107 (3)	0.0180 (4)	−0.0002 (2)	0.0043 (3)	−0.0002 (3)
N3A	0.0130 (3)	0.0091 (3)	0.0198 (4)	−0.0011 (2)	0.0053 (3)	0.0010 (3)
N4A	0.0149 (4)	0.0120 (3)	0.0268 (5)	−0.0015 (3)	0.0091 (3)	−0.0011 (3)
N5A	0.0152 (4)	0.0103 (3)	0.0297 (5)	−0.0001 (3)	0.0101 (3)	0.0001 (3)
C5A	0.0131 (4)	0.0102 (3)	0.0143 (4)	−0.0008 (3)	0.0021 (3)	0.0000 (3)
C6A	0.0132 (4)	0.0102 (3)	0.0156 (4)	−0.0014 (3)	0.0034 (3)	0.0009 (3)
N1B	0.0120 (3)	0.0091 (3)	0.0172 (4)	0.0013 (2)	0.0036 (3)	−0.0021 (3)
N2B	0.0129 (3)	0.0115 (3)	0.0198 (4)	0.0000 (3)	0.0055 (3)	−0.0004 (3)
N3B	0.0118 (3)	0.0097 (3)	0.0217 (4)	0.0009 (2)	0.0050 (3)	−0.0017 (3)
N4B	0.0132 (3)	0.0134 (3)	0.0186 (4)	0.0002 (3)	0.0043 (3)	−0.0025 (3)
N5B	0.0131 (3)	0.0121 (3)	0.0271 (4)	−0.0007 (3)	0.0062 (3)	−0.0037 (3)
C5B	0.0139 (4)	0.0105 (3)	0.0127 (4)	0.0004 (3)	0.0027 (3)	−0.0006 (3)
C6B	0.0127 (4)	0.0106 (3)	0.0150 (4)	0.0010 (3)	0.0020 (3)	−0.0012 (3)

### Geometric parameters (Å, º)

**Table:**

O1—C1	1.2599 (10)	N4A—H2N4	0.904 (15)
O2—C2	1.2608 (10)	N5A—C6A	1.3272 (12)
O3—C3	1.2490 (10)	N5A—H1N5	0.934 (15)
O4—C4	1.2622 (10)	N5A—H2N5	0.922 (15)
C1—C2	1.4582 (12)	N1B—C6B	1.3517 (11)
C1—C4	1.4642 (11)	N1B—C5B	1.3798 (11)
C2—C3	1.4690 (12)	N1B—H2N1	0.873 (15)
C3—C4	1.4627 (12)	N2B—C5B	1.3092 (11)
N1A—C6A	1.3559 (11)	N2B—N3B	1.3947 (10)
N1A—C5A	1.3805 (11)	N3B—C6B	1.3313 (11)
N1A—H1N1	0.913 (16)	N3B—H2N3	0.965 (15)
N2A—C5A	1.3127 (11)	N4B—C5B	1.3719 (12)
N2A—N3A	1.4020 (11)	N4B—H3N4	0.919 (14)
N3A—C6A	1.3280 (11)	N4B—H4N4	0.867 (13)
N3A—H1N3	0.937 (15)	N5B—C6B	1.3305 (11)
N4A—C5A	1.3513 (12)	N5B—H3N5	0.922 (15)
N4A—H1N4	0.887 (16)	N5B—H4N5	1.001 (15)
			
O1—C1—C2	134.67 (8)	N2A—C5A—N4A	126.16 (8)
O1—C1—C4	135.90 (8)	N2A—C5A—N1A	111.86 (8)
C2—C1—C4	89.41 (6)	N4A—C5A—N1A	121.97 (8)
O2—C2—C1	134.74 (8)	N5A—C6A—N3A	125.85 (8)
O2—C2—C3	134.50 (8)	N5A—C6A—N1A	127.51 (8)
C1—C2—C3	90.75 (7)	N3A—C6A—N1A	106.64 (8)
O3—C3—C4	135.13 (8)	C6B—N1B—C5B	106.58 (7)
O3—C3—C2	135.81 (8)	C6B—N1B—H2N1	125.0 (10)
C4—C3—C2	89.06 (6)	C5B—N1B—H2N1	128.4 (10)
O4—C4—C3	134.24 (8)	C5B—N2B—N3B	103.72 (7)
O4—C4—C1	134.98 (8)	C6B—N3B—N2B	111.33 (7)
C3—C4—C1	90.76 (7)	C6B—N3B—H2N3	129.8 (9)
C6A—N1A—C5A	106.58 (7)	N2B—N3B—H2N3	118.2 (9)
C6A—N1A—H1N1	125.0 (10)	C5B—N4B—H3N4	116.6 (8)
C5A—N1A—H1N1	128.3 (10)	C5B—N4B—H4N4	113.4 (8)
C5A—N2A—N3A	103.37 (7)	H3N4—N4B—H4N4	113.5 (11)
C6A—N3A—N2A	111.54 (7)	C6B—N5B—H3N5	121.5 (9)
C6A—N3A—H1N3	128.4 (9)	C6B—N5B—H4N5	118.2 (9)
N2A—N3A—H1N3	119.8 (9)	H3N5—N5B—H4N5	120.3 (13)
C5A—N4A—H1N4	119.5 (10)	N2B—C5B—N4B	124.71 (8)
C5A—N4A—H2N4	119.9 (9)	N2B—C5B—N1B	111.71 (8)
H1N4—N4A—H2N4	117.5 (13)	N4B—C5B—N1B	123.58 (8)
C6A—N5A—H1N5	123.2 (9)	N5B—C6B—N3B	125.61 (8)
C6A—N5A—H2N5	116.8 (10)	N5B—C6B—N1B	127.73 (8)
H1N5—N5A—H2N5	119.6 (13)	N3B—C6B—N1B	106.64 (7)
			
O1—C1—C2—O2	−1.04 (18)	N3A—N2A—C5A—N4A	178.72 (9)
C4—C1—C2—O2	177.98 (11)	N3A—N2A—C5A—N1A	0.26 (9)
O1—C1—C2—C3	179.83 (10)	C6A—N1A—C5A—N2A	0.32 (10)
C4—C1—C2—C3	−1.15 (7)	C6A—N1A—C5A—N4A	−178.20 (8)
O2—C2—C3—O3	1.85 (19)	N2A—N3A—C6A—N5A	−179.82 (9)
C1—C2—C3—O3	−179.02 (11)	N2A—N3A—C6A—N1A	1.01 (10)
O2—C2—C3—C4	−177.99 (11)	C5A—N1A—C6A—N5A	−179.95 (9)
C1—C2—C3—C4	1.15 (7)	C5A—N1A—C6A—N3A	−0.80 (10)
O3—C3—C4—O4	−2.60 (18)	C5B—N2B—N3B—C6B	−1.43 (10)
C2—C3—C4—O4	177.24 (10)	N3B—N2B—C5B—N4B	−179.41 (8)
O3—C3—C4—C1	179.02 (11)	N3B—N2B—C5B—N1B	1.21 (10)
C2—C3—C4—C1	−1.14 (7)	C6B—N1B—C5B—N2B	−0.61 (10)
O1—C1—C4—O4	1.79 (19)	C6B—N1B—C5B—N4B	180.00 (8)
C2—C1—C4—O4	−177.21 (11)	N2B—N3B—C6B—N5B	179.79 (9)
O1—C1—C4—C3	−179.85 (11)	N2B—N3B—C6B—N1B	1.09 (10)
C2—C1—C4—C3	1.15 (7)	C5B—N1B—C6B—N5B	−178.98 (9)
C5A—N2A—N3A—C6A	−0.80 (9)	C5B—N1B—C6B—N3B	−0.32 (9)

### Hydrogen-bond geometry (Å, º)

**Table:**

*D*—H···*A*	*D*—H	H···*A*	*D*···*A*	*D*—H···*A*
N1*A*—H1*N*1···O4^i^	0.913 (16)	1.761 (16)	2.6677 (9)	171.3 (15)
N3*A*—H1*N*3···O4^ii^	0.937 (15)	1.746 (15)	2.6734 (10)	170.2 (14)
N4*A*—H1*N*4···O3^i^	0.887 (15)	2.003 (15)	2.8877 (10)	175.2 (13)
N4*A*—H2*N*4···N4*B*^iii^	0.904 (15)	2.565 (15)	3.3917 (12)	152.3 (12)
N5*A*—H1*N*5···N2*A*^iv^	0.933 (15)	2.105 (15)	3.0167 (11)	165.3 (13)
N5*A*—H2*N*5···O1^ii^	0.922 (15)	1.940 (15)	2.8621 (11)	177.7 (14)
N1*B*—H2*N*1···O2^v^	0.874 (15)	1.781 (15)	2.6485 (9)	171.8 (15)
N3*B*—H2*N*3···O2	0.965 (15)	1.706 (15)	2.6637 (10)	171.2 (14)
N4*B*—H3*N*4···O1^v^	0.920 (14)	2.065 (14)	2.9564 (10)	162.8 (12)
N4*B*—H4*N*4···O1^vi^	0.867 (13)	2.150 (13)	2.9954 (10)	164.9 (12)
N5*B*—H3*N*5···N2*B*^v^	0.923 (15)	2.159 (15)	3.0579 (11)	164.3 (12)
N5*B*—H4*N*5···O3	1.001 (16)	1.832 (15)	2.8293 (10)	174.2 (12)

Symmetry codes: (i) −*x*, −*y*+1, −*z*+1; (ii) *x*, −*y*+3/2, *z*−1/2; (iii) *x*−1, −*y*+3/2, *z*+1/2; (iv) −*x*, *y*−1/2, −*z*+1/2; (v) −*x*+1, *y*−1/2, −*z*+1/2; (vi) −*x*+1, −*y*+2, −*z*+1.
